# The effect of comorbidity and frailty on dietary intake and protein losses in end-stage kidney disease patients undergoing peritoneal dialysis

**DOI:** 10.1038/s41598-026-54701-y

**Published:** 2026-06-05

**Authors:** Haalah Shaaker, Simon Eaton, Andrew Davenport

**Affiliations:** 1 Division of Medicine, University College London, London, UK; 2 King Abdulaziz University, Jeddah, Saudi Arabia; 3 Great Ormond Street Institute of Child Health, University College London, London, UK; 4 Centre for Kidney & Bladder Health, Royal Free Hospital, University College London, London, UK

**Keywords:** Malnutrition, End stage kidney disease, Peritoneal dialysis, Nutrition intake, Sarcopenia, Frailty, Diseases, Endocrinology, Health care, Medical research, Nephrology

## Abstract

End-stage kidney disease (ESKD) patients are advised to follow restricted diets, increasing their risk of malnutrition. We aimed to determine whether co-morbidity and frailty affected dietary intake or protein losses in ESKD patients treated with peritoneal dialysis (PD). We assessed 42 PD patients (mean age 62 ± 16 years) for malnutrition, using the Global Leadership Initiative in Malnutrition (GLIM) criteria, 24-hour dietary recall, bioimpedance, and hand grip strength (HGS), and measured effluent dialysate nitrogen losses. The malnutrition rate was 43%, significantly associated with heart failure (HF) odds ratio (OR) 22.6 (95% Confidence Interval (CI):2.0–259.5, *p* = 0.012) and low appendicular lean mass index (ALMI) OR 0.13 (95% CI:0.03–0.55, *p* = 0.006). Sarcopenia was associated with lower dietary protein intake (DPI) (*p* < 0.05), and decreased protein losses (*p* < 0.05), whereas Frailty was associated with older age (*p* < 0.001), greater malnutrition risk (*p* < 0.05) and reduced HGS (*p* < 0.05). Diabetic patients also had higher malnutrition risk (*p* < 0.01). HF was associated with male gender (*p* < 0.05), and lower body fat (*p* < 0.05). Increasing numbers of older more co-morbid ESKD patients are being treated with PD, and those with sarcopenia, frailty, diabetes, and HF are at an increased risk of malnutrition. These findings highlight the need for more individualized nutritional assessments.

## Introduction

Patients with progressive chronic kidney disease (CKD) are advised to follow a low protein diet, but once established on dialysis, clinical guidelines, such as those from the Kidney Disease Outcomes Quality Initiative (KDOQI) then recommend a daily protein intake (DPI) of 1–1.2 g/kg/day along with a daily energy intake (DEI) of 25–30 kcal/kg/day to ensure nutritional adequacy for end-stage kidney failure (ESKD) patients treated with dialysis^1^. However dietary intake may be reduced in ESKD patients due to altered taste perception, dietary restrictions, inflammation that disrupts appetite-regulating neuropeptides, and nutrient losses during dialysis treatments^2–5^. In addition, appetite may be further affected in patients treated by peritoneal dialysis (PD) due to the physical presence of intra-abdominal dialysate and glucose absorption from the dialysate^6,7^.

Malnutrition is defined as poor nutritional status resulting from inadequate intake, leading to depletion of muscle and fat stores^8^. Assessment of nutritional status requires a comprehensive evaluation, including anthropometric measures (body mass index (BMI), muscle strength, and body composition), biochemical markers (albumin, C-reactive protein (CRP)), clinical history, physical examination, and dietary intake assessments such as dietary recalls and food frequency questionnaires^9^ More recently, the Global Leadership Initiative on Malnutrition (GLIM) has developed a global consensus for screening, assessment, diagnosis, and grading of malnutrition for ESKD patients treated with both PD and hemodialysis^10^.

Dialysis patients are at increased risk of sarcopenia, defined as progressive loss of muscle mass and strength, greater than that expected due to physiological aging, with a reported prevalence of 26–35%, and associated with older age, longer dialysis vintage and increased mortality^11,12^. Frailty on the other hand is a functional assessment, characterized by increased dependency and mortality,^13,14^.

Over time, the demographics of the ESKD dialysis population have changed, with greater numbers of older, more co-morbid patients now being offered treatment with PD. Similarly, with increasing use of automated peritoneal cyclers (APD) rather than the traditional continuous ambulatory peritoneal dialysis (CAPD), with potentially increased protein losses, making nutrition care plans even more critical than ever in older more co-morbid patients. As such we wished to evaluate the prevalence of malnutrition, dietary intake, and dialysate and urinary protein losses in PD patients, focusing on those with sarcopenia, frailty, diabetes and heart failure.

## Methods

### Study population and data collection

This prospective cross-sectional observational study recruited 42 adult PD patients. This study was designed as a pilot observational study, and therefore no formal power calculation was performed. Patients unable to provide accurate dietary recall, due to cognitive, impairment, physical disability or language barriers were excluded. All patients used glucose-based PD dialysates and 7.5% icodextrin (Baxter Health Care, Deerfield, Illinois, USA) for overnight exchanges with CAPD or daytime exchanges with automated continuous cycling PD (CCPD)^15^. Patients received standard multidisciplinary care, including routine support from renal dietitians.

### Study materials and procedure

#### Malnutrition assessment

Malnutrition was assessed using the GLIM criteria and the Malnutrition Universal Screening Tool (MUST)^16^. MUST scores were based on body mass index (BMI), weight loss, and insufficient food intake to identify malnutrition risk. Malnutrition was confirmed by the presence of one phenotypic (weight loss, low BMI) and one etiological criterion (inflammation, reduced dietary intake). Malnutrition severity was graded as moderate or severe based on phenotypic criteria, with data collected from patient interviews and medical records.

### Dietary intake

A 24-hour dietary recall was conducted by a single trained dietitian to estimate nutrient intake, and analysed using Nutritics nutritional analysis software (Nutritics, Swords, County Dublin, Ireland)^17^.

### Conditions of interest

The European Working Group on Sarcopenia (EWGSOP) criteria were used to define sarcopenia in non-Asians [hand grip strength (HGS) < 27 kg for men, < 16 kg for women, and appendicular lean mass index (ALMI) < 7 kg/m² for men, < 5.5 kg/m² for women], and the Asian Working Group for Sarcopenia (AWG) cut offs for Asian patients [HGS of < 28 kg and < 18 kg, and ALMI < 7.0 kg/m^2^ and < 5.7 kg/m^2^ for Asian men and women respectively]^18,19^. Frailty was identified using a clinical frailty score (CFS) ≥ 4 ^20^. Computerized medical records were reviewed to identify patients with diabetes (DM) defined according to the World Health Organization definition, and heart failure (HF) as a reduced cardiac ejection fraction on cardiac imaging using two-dimensional M mode transthoracic echocardiogram (Philips IE33; Philips Medical Systems, Eindhoven, the Netherlands), along with additional variables including age, weight, ethnicity, and PD prescriptions.

### Anthropometry

Body composition was measured using multifrequency bioelectrical impedance analysis (BIA), following a standard protocol, after patients had voided and peritoneal dialysate drained (InBody 770, Seoul, Sout Korea)^21^. Bioimpedance equipment was regularly serviced and calibrated. To compare patients the appendicular lean mass index (ALMI) was calculated by dividing appendicular lean mass (ALM) by height squared^22^.

### Biochemical investigations

Computerized laboratory results, including 24-hour urinary protein loss, serum albumin, C-reactive protein (CRP), and hemoglobin, all measured by an accredited United Kingdom (UK) laboratory, were extracted from medical records at the time of patient review. The normalized protein nitrogen accumulation rate (nPNA) was estimated based on combined 24-hour urinary and dialysate urea losses^23,24^. and dialysis adequacy determined by weekly urea urinary and peritoneal clearance (Kt/V) using standard formulae^25^.

### Physical assessment

The Duke Activity Status Index (DASI) questionnaire was used to assess functional capacity^26^, and HGS measured with a Grip-D dynamometer (Takei 5401 Hand Grip Digital Dynamometer, Shinagawa-ku, Tokyo, Japan) to determine muscle power, with patients instructed according to the manufacturer’s instructions, with the maximum HGS of three measurements recorded^27^.

### Dialysate nitrogen loss

24-hour effluent dialysate samples were collected and stored at -50 °C until analysis. Total nitrogen concentration measured in batches (Antek MultiTek nitrogen analyzer, Cape Town, South Africa). As most laboratory techniques for measuring urea also detect ammonia, we employed a colorimetric assay based on the method described by (Rahmatullah. M & Boyde, Hong Kong)^28^, with slight modifications and a microplate reader to accurately quantify only urea content. Briefly, the assay involves the enzymatic conversion of urea into a colored product proportional to the urea concentration. The absorbance of the resulting solution was measured using a Tecan NanoQuant Infinite 200 microplate reader equipped with i-Control 2.0 software (Tecan Group Ltd., Männedorf, Switzerland)^29^. Non-urea nitrogen (NUN) loss was then calculated by subtracting urea nitrogen from the total nitrogen concentration. Protein content in the dialysate from NUN was calculated by multiplying NUN (g) by a factor of 6.25 to estimate protein equivalents^1^. Urinary protein losses were analysed in the UK accredited clinical laboratory (Cobas Integra, Roche Molecular Systems, Inc, Burgess Hill, Sussex, UK).

### Ethical approval

The study was approved by the UK National Health Service (NHS) Research Authority Ethics Service (NRES) (EDGE 158772, IRAS29331). In keeping with the Helsinki accord, all patients provided written informed consent. All methods were carried out in accordance with relevant guidelines and regulations, and in line with NRES protocols, all data were anonymized.

### Statistical analysis

Data normality was assessed using skewness and kurtosis. Categorical variables were analysed using chi-squared tests, and numerical data presented as mean ± SD or median [interquartile range (IQR)]. Comparisons were made using independent t-tests or Mann-Whitney U tests. Paired t-tests or Wilcoxon signed-rank tests were used for within-group comparisons, with appropriate post-hoc testing. Univariate correlations were evaluated with Pearson or Spearman correlation coefficients. Receiver operator curves (ROC) were drawn to identify variables associated with malnutrition, and variables associated with malnutrition on ROC analysis were entered into a step backward multivariable logistic regression model to determine which variables were independently associated with malnutrition. If required variables were log transformed to improve normality distribution. Variables were then removed or retained in the model if the 95% confidence intervals for the estimate did not include zero or there was an improvement in model fit (as demonstrated by the − 2log likelihood). Models were checked for collinearity and variable inflation factor to prevent overloading and checked for collider bias. Analyses were performed with IBM SPSS Statistics Version 29 (IBM, Armonk, New York, USA), with significance taken at *p* < 0.05.

## Results

### Demographics and nutritional assessment of peritoneal dialysis patients

#### Patient demographics and malnutrition

We studied 42 multiethnic PD patients out of a population of 45 patients, one patient was unable to provide a dietary history due to language barrier, and two patients declined to take part. All patients received adequate dialysis treatment by achieving UK target amounts of weekly urea clearance (Kt/V)^30^.

The mean age of the study cohort was 62 ± 16 years, with similar numbers treated with CAPD (43%) and CCPD (43%), and the remainder treated with APD (14%), and a median dialysis vintage of 12 months (IQR 3.1–23.3). Malnutrition risk assessed using the GLIM criteria, revealed that 69% were at high risk, 43% had confirmed malnutrition, and 14% had severe malnutrition (Table 1).

**Table 1:** Demographic, malnutrition assessment, and nutrient loss of peritoneal dialysis participants with and without sarcopenia, frailty, diabetes and heart failure.

	All	Sarcopenia		Frailty		Diabetes		Heart Failure	
	*n* = 42	Sarcopenic n = 9 (21%)	Non- Sarcopenic n = 33 (79%)	Frail n = 19 (45%)	Non-Frail = 23 (55%)	Diabetic n = 18 (43%)	Non-Diabetic n = 24 (57%)	Heart Failure n = 16 (38%)	No Heart Failure n = 26 (62%)
Demographic									
Age (years)	62 ± 16	66 ± 16	61 ± 16	71 ± 11***	55 ± 16	56.56 ± 15.80*	66.38 ± 15.12	64 ± 18	61 ± 15
Males, n (%)	24 (57)	6 (67)	18 (45)	13 (68)	11 (47)	10 (55.5)	14 (58.3)	13 (81) *	11 (42)
White, n (%)	18 (43)	1 (11)	17 (52)	10 (53)	8 (35)	5 (28)	13 (54)	8 (50)	10 (38)
Black, n (%)	13 (31)	2 (22)	11 (33)	3 (19)	10 (43)	5 (28)	8 (33)	4 (25)	9 (35)
Asian, n (%)	10 (24)	5 (56) **	5 (15)	5 (26)	5 (22)	7 (39)	3 (13)	3 (19)	7 (27)
Others, n (%)	1 (2)	1 (11)	0	1 (2)	0	1 (5)	0	1 (6)	0
Dialysis Vintage (Months)	12.7 (3.1–23.3)	15.2 (3.07–39.53)	12.17 (3.57–22.8)	14.2 (8.1–22.3)	11.17 (3.07–24.33)	11.65 (3.07–22.81)	14.17 (3.32–23.3)	14.2 (9.11–41.57)	11.67 (3.07–22.55)
Residual kidney function (ml/min)	5.2 (1.6–8.3)	1.6 (0–8.6)	5.6 (2.6–8)	6.2 (3.8–8.9)	2.8 (1.1–7.7)	4.5 (0.7–7.7)	6 (2.6–9)	7.3 (1.6–9.7)	5 (2.2–7.4)
KtVurea	2.04 (1.69–2.35)	2.06 (1.56–2.26)	2.02 (1.72–2.5)	2.25 (2.1–2.51) **	1.78 (1.6–2.12)	2.15 (1.48–2.53)	2.02 (1.76–2.27)	2.15 (1.77–2.45)	2 (1.65–2.34)
Malnutrition assessment									
At high-risk vs. low risk	29 (69)	9 (100)	20 (60)	16 (84) *	13 (56)	17 (94) **	12 (50)	12 (75)	17 (75)
Malnourished vs. well nourished	12 (43)	7 (78) ***	5 (15)	7 (37)	5 (22)	6 (33)	6 (25)	8 (50) *	4 (15)
Severely vs. moderately malnourished	8 (14)	7 (78) ***	1 (3)	5 (26)	3 (13)	5 (27)	3 (12)	6 (37) *	2 (8)
Protein losses									
Total nitrogen in dialysate (g/day)	2.65 (1.64–4.12)	1.87 (1.39–2.26)	2.86 (1.82–4.31)	2.24 (1.56–3.98)	2.85 (1.70–4.19)	2.67 (1.98–4.73)	2.01 (1.59–3.78)	2.89 (1.64–4.36)	2.47 (1.64–3.79)
Non-Urea Nitrogen (g/day)	0.69 (0.51–1.58)	0.59 (0.26–0.66) *	1.06 (0.56–1.79)	0.75 (0.48–1.46)	0.69 (0.52–1.77)	0.69 (0.51–2.18)	0.75 (0.51–1.56)	0.67 (0.42–1.72)	0.99 (0.55–1.45)
Protein in urine (g/day)	0.33 (0.1–1.35)	0.22 (0.03–0.94)	0.37 (0.18–1.46)	0.35 (0.17–1.43)	0.28 (0.06–1)	0.33 (0.07–1.36)	0.35 (0.11–1.35)	0.28 (0.06–1.33)	0.51 (0.18–1.77)
Protein loss in dialysate (g/day)	4.29 (3.16–9.9)	3.66 (1.65–4.13) *	6.64 (3.48–11.2)	4.67 (2.98–9.15)	4.29 (3.26–11.08)	4.29 (3.16–13.64)	4.70 (3.19–9.76)	4.18 (2.63–10.78)	6.19 (3.43–9.08)
Glucose Absorption (mmol/day)	73 (-17.9 to 247.9)	64.1 (-38.15 to 470.3)	85.4 (-16.3 to 230.95)	64.1 (-32.8 to 400)	127.3 (-11.2 to 240.1)	72.2 (-38.15 to 255.4)	85.4 (-12.2 to 254.12)	113.15 (-33.3 to 289)	72.2 (15.1 to 247.8)

P<0.05*, P<0.01**, P<0.001***.

**Fig. 1:**
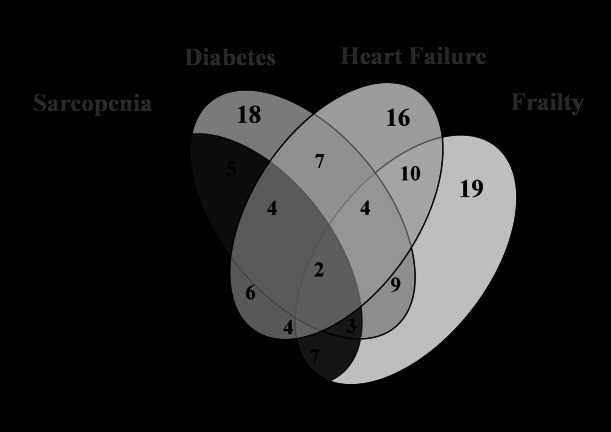
Venn diagram of additional comorbidities. This shows the overlap of sarcopenia, frailty, diabetes (DM), and heart failure (HF) among peritoneal dialysis (PD) patients.

Almost 50% of patients had DM and frailty, with fewer patients classified as having sarcopenia or HF (Table 1). There were numerous overlaps between these conditions (Fig. 1). Sarcopenia was significantly associated with frailty (*p* = 0.026) and HF (*p* = 0.049). Only 26% of patients had no additional co-morbidity.

### Nutritional assessment

The majority of patients had a BMI in the overweight range (26.7 ± 4.9 kg/m²), and ALMI above the cut off for sarcopenia. Serum albumin and CRP values were within the laboratory reference range for most patients. Hemoglobin values were generally low but remained within the recommended targets for ESKD patients (Table 2). Additionally, 17% of participants (*n* = 7) followed a vegetarian diet, with majority (71%) being of Asian ethnicity (*n* = 5).

**Table 2:** Nutritional assessment of peritoneal dialysis participants with and without sarcopenia, frailty, diabetes and heart failure.

	All	Sarcopenia		Frailty		Diabetes		Heart Failure	
	*n* = 42	Sarcopenic n = 9 (21%)	Non- Sarcopenic n = 33 (79%)	Frail n = 19 (45%)	Non-Frail = 23 (55%)	Diabetic n = 18 (43%)	Non-Diabetic n = 24 (57%)	Heart Failure n = 16 (38%)	No Heart Failure n = 26 (62%)
Dietary Intake									
Energy (Kcal/kg)	17.04 (13.5–20.8)	19.04 (15.52–26.7)	16.31 (13.23–19.5)	17.14 (13.24–20.71)	16.78 (13.35–24.6)	17.26 (13.31–19.7)	16.78 (13.58–23.7)	15.39 (13.57–20.5)	17.26 (13.31–21.9)
Protein (g/kg)	0.75 (0.55- 1)	0.68 (0.52–0.76)	0.81 (0.56–1.03)	0.7 (0.53–1.01)	0.79 (0.55–1.04)	0.68 (0.53–1)	0.84 (0.57–1.02)	0.72 (0.56–1)	0.82 (0.56–1.1)
Energy (Kcal)	1133 (937–1456)	1197 (979–1571)	1099 (930–1462)	1070 (885–1247)	1134 (952–1591)	1083 (949–1484)	1133 (929–1445)	1070 (943–1298)	1165 (931–1761)
Protein (g)	50.7 (38.1–74.1)	37.5 (31.8–5) *	55 (43.2–88.4)	48.5 (32.1–56.6)	62.6 (38.7–92.4)	46.7 (33.5–63.7)	54.9 (42.3–92.3)	47.17 (37.5–61.6)	54.7 (37.1–93.7)
nPNA (g/kg)	0.91 (0.77–1.08)	0.86 (0.62–1.05)	0.92 (0.8–1.12)	0.93 (0.84–1.18)	0.85 (0.73–0.99)	0.86 (0.69–1.05)	0.93 (0.82–1.12)	0.92 (0.84–1.02)	0.9 (0.74–1.09)
Biochemical results									
Albumin (g/L)	37.33 ± 5.03	34.67 ± 5.05	38.06 ± 4.84	36 ± 5.71	38.43 ± 4.20	37.06 ± 4.6	37.54 ± 5.39	37.94 ± 4.75	36.96 ± 5.24
CRP (mg/L)	3.00 [1–8.25)	4 [2–19)	3 [1–8.5)	2 [1–6)	3 [2–9)	3 [2–11.25)	2.5 [1–7.5)	2 [1–4.75)	3.5 [1.75–12)
Hemoglobin (g/dl)	11.5 ± 1.2	12 ± 0.6 *	11.3 ± 1.2	11.6 ± 1.2	11.4 ± 1.2	11.6 ± 1.1	11.2 ± 1.2	11.8 ± 1	11.3 ± 1.2
Anthropometry									
BMI (kg/m2)	26.7 ± 4.9	23.6 ± 2.6 **	27.5 ± 5	25.8 ± 4.5	24.3 ± 5.2	27.4 ± 5.6	26.1 ± 4.3	24 ± 5	27.7 ± 4.5
BFP (%)	23.7 (18.3–31)	36.6 (31.5–38.1)	32.1 (24.9–42.1)	24 (29–36.7)	24 (17–35)	35.2 (28.8–43)	33.2 (23.2–38.7)	30.8 (22.3–36.7) *	34.5 (30.3–43.3)
ALM (kg)	19.1 (15–27)	15.18 (13–19.8) *	19.7 (16–27.8)	19.56 (13.7–21.7)	18.74 (15–27.2)	19.15 (14.7–21.6)	20.19 (14.9–27.2)	19.8 (15.6–27)	19.1 (14.5–24.8)
ALMI (kg/m2)	7.4 ± 1.6	6.1 ± 0.7 **	7.7 ± 1.6	7.3 ± 1.7	7.4 ± 1.5	7.3 ± 1.8	7.4 ± 1.5	7.3 ± 1.6	7.4 ± 1.6
ECW/TBW	0.40 ± 0.01	0.4 ± 0.01	0.4 ± 0.02	0.41 ± 0.02 **	0.39 ± 0.01	0.40 ± 0.01	0.39 ± 0.01	0.40 ± 0.01	0.4 ± 0.02
Physical assessment									
DASI	25.2 (15.4–38.5)	15.4 (10.9–29.5)	28.7 (15.4–42)	15.4 (11.7–23.4) ***	32 (21.5–42.7)	18.98 (9.3–35.5)	28.7 (15.5–41)	17.4 (12.7–37.4)	28.7 (15.4–42.1)
HGS (kg)	23 (25–31)	19.4 (13.1–21.2) *	26.5 (19.4–33.3)	20.5 (15.9–24.3) *	29.2 (20.3–33.9)	20.80 (15.3–27.9)	26 (20.3–33.9)	23.4 (20.6–33.9)	22.8 (15.8–29.9)

P<0.05*, P<0.01**, P<0.001***.

nPNA, normalized protein nitrogen appearance rate. CRP, C-reactive protein. BMI, body mass index. BFP, body fat percentage. ALM, Appendicular Lean Mass. ALMI, Appendicular Lean Mass Index. ECW/TBW, extracellular Water/Total Body Water. DASI, duke activity score index. HGS, Hand grip strength.

The DEI was 17.04 kcal/kg/day (IQR 13.5–20.8), and the DPI was 0.75 g/kg/day (IQR 0.55–1.0) (Table 2). In regard to mineral intake, the median intake was 1276 mg/day (IQR 828–1630) for sodium, 1737 mg/day (IQR 1468–2173) for potassium, and 722 mg/day (IQR 560–1049) for phosphorus.

### Dietary targets in peritoneal dialysis patients

**Fig. 2:**
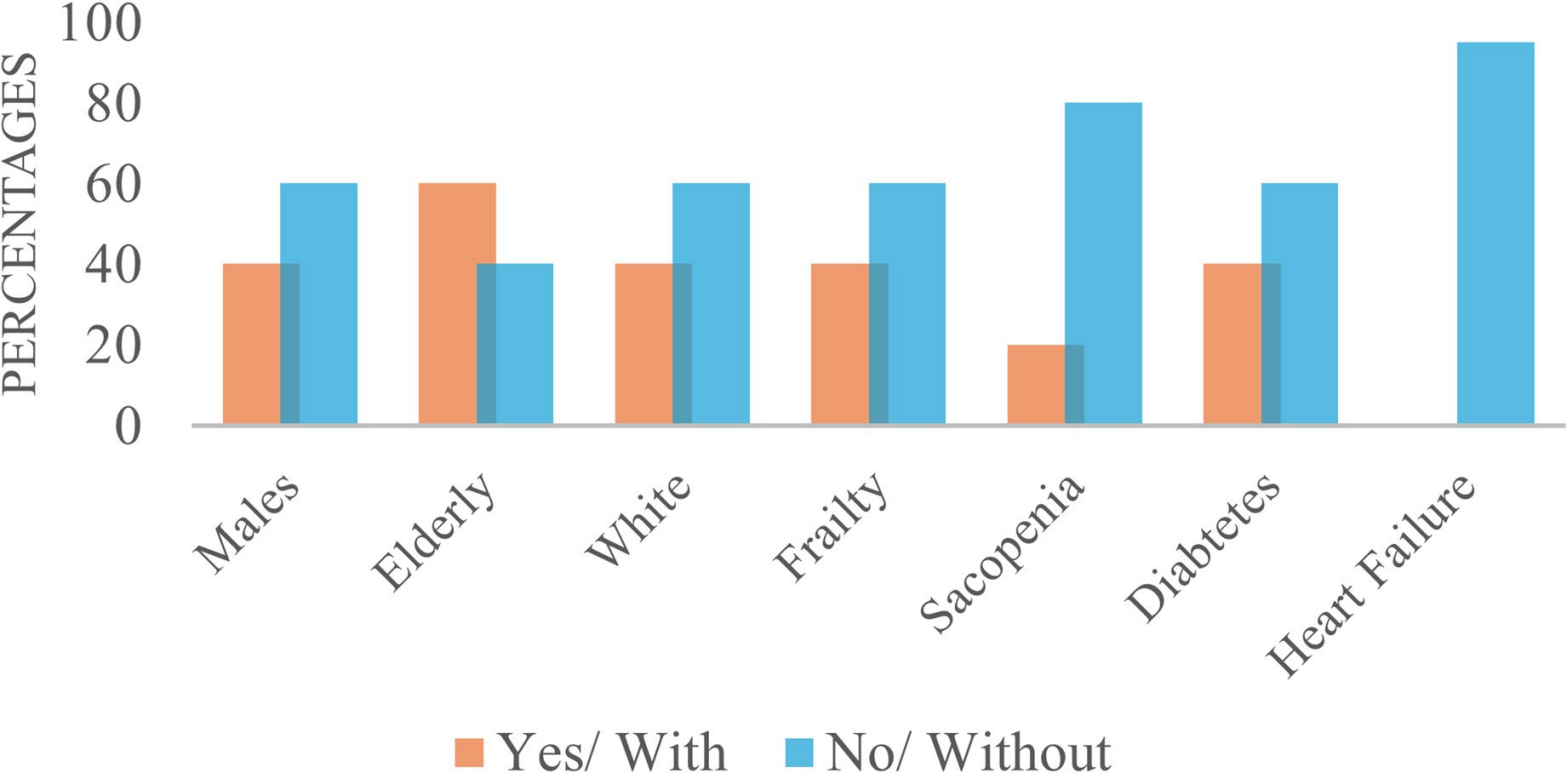
Demographic and clinical characteristics of participants achieving the recommended daily energy intake (DEI ≥ 25 kcal/kg) and daily protein intake (DPI ≥ 1 g/kg) targets. Comparison includes gender (male vs. female), age (elderly ≥ 65 years vs. younger < 65 years), ethnicity (White vs. other), and comorbidities (frailty, sarcopenia, diabetes, and heart failure).

Based on KDOQI recommendations (energy intake ≥ 25 kcal/kg/day and DPI ≥ 1 g/kg/day, then only 12% of patients met both targets, 17% achieved adequate energy intake, and one-third met protein recommendations. As shown in Fig. 2, over half of those achieving the targets were female and of non-White ethnicity. Interestingly, this group was predominantly aged ≥ 64 years and did not have sarcopenia, frailty, diabetes, or heart failure. High protein and calorie supplements were prescribed to 5 patients, 80% (*n* = 4) of them were in the older adult group.

### Peritoneal dialysis patients with and without sarcopenia

Sarcopenic patients were predominantly Asian with greater frailty, and had significantly lower dialysate protein loss but with no significant differences in dialysis treatment, or physical activity (Table 1). All sarcopenic patients were classified as high risk for malnutrition, compared to 60% of non-sarcopenic patients. Sarcopenia was significantly associated with both the presence (X²=13.6, *p* < 0.001) and severity (X²=25.7, *p* < 0.001) of malnutrition. Sarcopenic patients had significantly lower DPI, despite having slightly higher calorie intake (Table 2).

### Peritoneal dialysis patients with and without frailty

Frail PD patients were older and received more dialysis clearance (weekly Kt/V), but with no significant differences in dialysis protein losses or residual kidney function (Table 1). Frailty was associated with a significantly higher risk of malnutrition although the prevalence of confirmed malnutrition was similar between the groups. Frail patients had lower HGS, with greater fluid overload as assessed by the extracellular water to total body water ratio (ECW/TBW) and reduced functional capacity assessed by (DASI) (Table 2).

### Peritoneal dialysis patients with and without diabetes

Diabetic patients were younger and at higher risk of malnutrition, with no significant differences in dialysis adequacy or protein losses (Table 1). There were also no significant differences in BMI, body composition, activity scores, HGS, or dietary protein or calorie intake (Table 2).

### Peritoneal dialysis patients with and without heart failure

Most HF patients were male and more severely malnourished than those without HF (Table 1). HF patients had significantly lower percentage body fat, although muscle mass and physical activity levels were similar. They consumed slightly fewer calories and protein (Table 2), with significantly lower dietary intake of trans-fatty acids (0.077 [0.01–0.4] vs. 0.34 [0.11–0.64] g/day, *p* = 0.009) and calcium (313 [269–473] vs. 553 [332–835] mg/day, *p* = 0.023).

### Correlation between dietary intake and measured dialysate protein losses

When comparing dietary protein intake (DPI, g/kg) estimated from 24-hour dietary recall with daily protein nitrogen appearance (PNA, g/kg), a significant difference was observed between the 24-hour recall estimates and PNA (57.8 ± 25.7 vs. 67.3 ± 24.4 g/day, *p* = 0.027).

Among the PD cohort, the median protein loss in dialysate was 4.29 g/day (IQR 3.16–9.9), while the median NUN loss was 0.69 g/day (IQR 0.51–1.58) (Table 1). However, no association was found between energy or protein intake assessed by 24-hour recall and protein loss (g/day) or NUN loss (g/day) in dialysate. For instance, the correlation between Energy intake (kcal/day) and PD protein loss was (*r* = -0.073, *p* = 0.664) and with NUN loss (*r* = -0.073, *p* = 0.664). The correlations between protein intake (g/day) and PD protein loss (*r* = 0.287, *p* = 0.080) and NUN loss (g/day) in dialysate (*r* = 0.287, *p* = 0.080), were also non-significant.

Similarly, there were no correlations between protein intake determined by PNA (g/day) and protein loss (g/day) (*r* = 0.078, *p* = 0.639) and NUN loss (g/day) ((*r* = 0.078, *p* = 0.639) in the dialysate. In addition, there were no significant differences in protein loss between PD modalities.

**Fig. 3:**
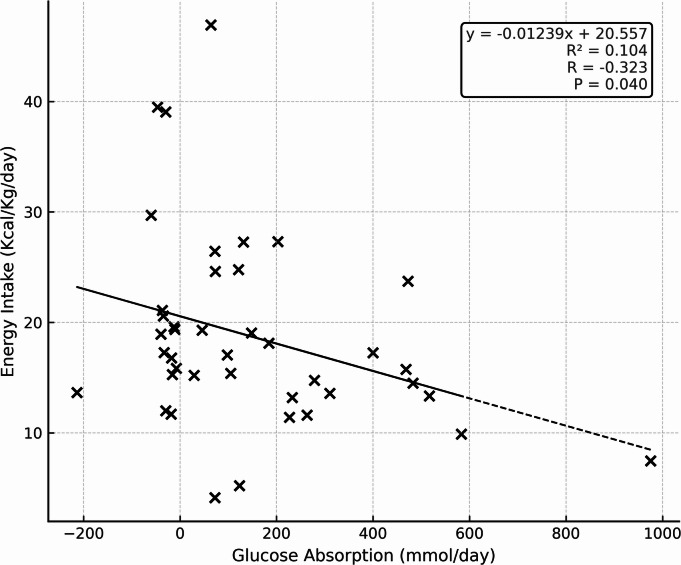
Association between peritoneal glucose absorption and daily energy intake (DEI) in peritoneal dialysis patients. Scatter plot showing a significant relationship between higher glucose absorption (mmol/day) and lower energy intake (kcal/kg/day).

However, greater residual kidney function was associated with higher dietary protein intake (*r* = 0.352, *p* = 0.033), whereas patients with higher daily peritoneal glucose absorption from the dialysate were significantly associated with lower dietary energy intake (Fig. 3).

### Receiver operator curve and multivariable logistic regression analyses

The ROC analysis identified sarcopenia (AUC = 0.78, 95% CI: 0.60–0.97, *p* = 0.003) and HF (AUC = 0.73, 95% CI: 0.55–0.91, *p* = 0.014) as significant factors associated with malnutrition in PD patients (Table 3), whereas ALMI (AUC = 0.15, 95% CI: 0.03–0.26, *p* < 0.001). and HGS (AUC = 0.33, 95% CI: 0.16–0.5, *p* = 0.050), key determinants of sarcopenia, were inversely associated with malnutrition. In contrast, frailty and diabetes had no significant association with malnutrition. There was no association found between either total nitrogen or NUN loss in dialysate and malnutrition.

**Table 3:** Area under the receiver operating characteristic (ROC) curve of factors associated with malnutrition in peritoneal dialysis patients.

Test result variable(s)	Area	Std. error	*P* value	Asymptotic 95% confidence interval	
				Lower bound	Upper bound
Sarcopenia	0.784	0.094	0.003	0.599	0.968
Frailty	0.611	0.100	0.268	0.415	0.808
Diabetes	0.538	0.104	0.717	0.334	0.741
Heart failure	0.726	0.092	0.014	0.545	0.907
ALMI (kg/m2)	0.146	0.059	0.000	0.030	0.261
HGS (kg)	0.332	0.086	0.050	0.164	0.500
Nitrogen in dialysate (g/day)	0.417	0.096	0.387	0.229	0.605
NUN in dialysate (g/day)	0.395	0.094	0.265	0.210	0.580

*P*<0.05, *P*<0.01, *P*<0.001.

ALMI, Appendicular Lean Mass Index. HGS, Hand Grip Strength. NUN, Non-Urea Nitrogen.

A step backward multivariable logistic regression model was then generated using all variables with a *p* < 0.1 associated with malnutrition, and after adjusting for collider bias and collinearity, only the presence of HF (OR 22.6, 95% CI: 2.0–259.5, *p* = 0.012) and lower ALMI (OR 0.13, 95% CI: 0.03–0.55, *p* = 0.006) remained independently associated with malnutrition (Table 4).

**Table 4:** Multiple logistic regression analysis of factors associated with malnutrition in peritoneal dialysis patients: step 5 of backward regression (Nagelkerke R² = 0.668).

	B	S.E.	Wald	Odds Ratio	95% CI		*P* value
					Lower	Upper	
Heart failure	3.116	1.246	6.254	22.564	1.962	259.503	0.012
ALMI	-2.041	0.740	7.612	0.130	0.030	0.554	0.006

P<0.05*, P<0.01**, P<0.001***.

ALMI, Appendicular Lean Mass Index.

## Discussion

In this study, over half of the PD cohort were identified as being at high risk of malnutrition according to GLIM criteria, with 43% meeting the threshold for confirmed malnutrition. These findings are consistent with those of a study from China^31^, which reported a malnutrition prevalence of 35% among 1057 PD patients. Although the patients living in China were on average younger and all treated by CAPD, the similar findings between the two studies underscore the high prevalence of malnutrition in geographically different PD populations. In our study, the average DEI and DPI were both below the KDOQI-recommended targets. These findings are consistent with previous studies reporting insufficient dietary intake in PD patients^32,33^.

Only a minority of our PD population met the current energy and protein targets advised by clinical consensus, and these were primarily individuals without additional comorbidities or conditions including DM, HF, frailty and sarcopenia. This highlights the importance of nutritional screening and tailored nutritional management for patients with comorbidities and muscle wasting^34^. Perhaps unexpectedly, older participants in our cohort were more likely to meet nutritional targets, most likely due to the provision of high-calorie and protein supplements and structured meals in nursing home settings, whereas younger participants were more prone to meal skipping, often due to their busy schedules.

Previous studies reporting on sarcopenia and frailty, particularly those from non-UK populations, identified several factors including older age, male gender, low BMI, and malnutrition as risk factors for sarcopenia^34^. In the current study, we found that all patients with sarcopenia were at high risk of malnutrition, with the majority being severely malnourished and frail. Furthermore, multivariable logistic regression analysis confirmed a significant association between sarcopenia and malnutrition, identifying low ALMI as a specific risk factor. These findings reinforce the critical link between sarcopenia and malnutrition in this population. Although the correlation between DPI and protein loss in dialysate was weakly positive (*r* = 0.287, *p* = 0.080), both DPI and dialysate protein loss were lower in patients with sarcopenia. These findings suggest a potential association between dietary protein intake and the nutritional status of sarcopenic patients^35^. Additionally, our study investigated sarcopenia in different ethnic groups, finding a significant increase in sarcopenia in patients of Asian ethnicity, possibly due to limited protein sources associated with vegetarian and vegan diets.

In terms of frailty, our findings somewhat differ from an earlier study from Hong-Kong, which identified that frail patients were older, malnourished, and had greater micro-inflammation^36^. Whereas our frail patients did not have raised CRP or reduced serum albumin and had similar dietary protein and calorie intake compared to non-frail patients, but had lower muscle power, and were less physically active, suggesting that dietary intake alone does not influence physical inactivity in frail patients.

We also compared dietary intake in diabetics, and in keeping with reports in pre-dialysis chronic kidney disease (CKD) patients we also found no significant differences in DEI and DPI between diabetic and non-diabetic patients despite the additional dietary restrictions imposed on diabetic patients^37^.

A retrospective study from the US noted poor nutritional status among patients with both chronic heart failure (CHF) and CKD^38^. Our study compared PD patients with and without a reduced cardiac ejection fraction and found that half of the HF group were malnourished, mostly classified with severe malnutrition. Logistic regression analysis further confirmed HF as an independent risk factor for malnutrition. Although there were no differences in body composition or DPI and DEI, HF patients consumed lower amounts of trans-fat and calcium, possibly reflecting adherence to dietary recommendations aimed at limiting whole-fat dairy products and other fried foods, which may have inadvertently led to a reduced calcium intake.

Protein losses during dialysis treatments are well recognized, and recent advancements have highlighted the magnitude of these losses. A UK study found greater sessional protein, urea, and nitrogen losses with hemodialysis compared to PD, although weekly losses were similar^39^. Our study found no correlations between self-reported energy and protein intake and dialysate protein or NUN losses. Dietary protein intake estimated from 24-hour recalls was lower than calculated PNA, likely because the PNA equation accounts for insensible protein losses via stool, skin, and urine, which are not captured by dietary recall methods.

We also observed that PD patients with higher urine output tended to have greater DPI consumption, supporting the notion that residual kidney function influences dietary habits^40^. This may be explained by better clearance of uremic toxins, leading to fewer uremic symptoms such as anorexia and reduced appetite, thereby allowing higher protein intake. As patients without residual kidney function generally require the prescription of higher glucose dialysates to achieve adequate ultrafiltration, then this may potentially lead to increasing glucose absorption from the dialysate, and so reduce appetite due to the satiety induced by glucose calorie absorption^7^. Our results support this contention, demonstrating a significant correlation between higher glucose absorption and decreased dietary energy intake.

This study has both strengths and limitations that are worth acknowledging. Although we report associations between various aspects of nutritional intake, body composition, muscle strength, physical activity and additional co-morbidities in this cross-sectional analysis we cannot attribute causality. The relatively small sample size should be considered when interpreting the findings, as it may limit statistical power and generalizability, although it reflects typical PD cohort sizes in European dialysis units. Previous studies have highlighted inter-observer differences in obtaining dietary histories and measurement of both HGS and with bioimpedance measurements of body composition, particularly when peritoneal dialysate was not drained from the abdomen, or when electrodes had to be individually sited. So, to minimise data collection errors, a single trained dietitian investigator conducted this study, and bioimpedance equipment was chosen that standardized electrode placement. Diet recall can be affected by memory lapses, so we excluded patients with dementia, learning disorders and a history of previous stroke and vascular disease on cerebral imaging, and cases with a language barrier. As such, we acknowledge the sample size of patients completing food recalls, yet this was balanced between three main ethnic groups, but there may still be inherent patient recall bias. To limit errors in re-call, self-reported dietary intake was limited to the previous 24 h, as other studies have highlighted the difficulty that elderly co-morbid patients have in accurately recalling or documenting for longer time periods. The dietitian investigator provided detailed guidance to patients on dietary recall procedures. Dietary recall was performed at the same time as peritoneal dialysate collection to ensure temporal alignment of measurements. Nutritional data were exported directly from Nutritics software to an Excel spreadsheet to avoid recording errors. We measured total nitrogen in effluent peritoneal dialysate, which includes not only proteins and urea, but also small peptides, nucleotides, amino acids, organic acids, uric acid, nitrates and nitrites, and as such our measurements could potentially overestimate losses due to the small amounts of additional organic acids, urate and nitrates and nitrites in the spent dialysates.

## Conclusion and Implications

Our study indicated that nearly half of our multiethnic ESKD PD patients were malnourished, with malnutrition significantly associated with both sarcopenia and HF. Most patients failed to meet daily energy and protein intake targets. Those with additional comorbidities, including sarcopenia, frailty, DM, and HF, were at significantly higher risk of malnutrition and exhibited poorer nutritional parameters compared to those without these conditions. Furthermore, there was considerable overlap between these conditions. Despite these findings, overall dietary intake did not significantly differ across groups, except for lower protein intake in sarcopenic patients and reduced calcium and trans-fatty acid intake in those with HF.

Our findings emphasise the importance of screening for malnutrition in ESKD PD patients, particularly those with heart failure and other co-morbidities. As many patients failed to achieve currently recommended dietary protein intake targets, future prospective studies are required to determine whether achieving these targets would reverse malnutrition, or whether additional interventions, including exercise programmes would be required.

## Data Availability

The study data are stored on a secure UCL server and may be made available by the corresponding author (H.S.) upon reasonable request, subject to appropriate approvals. All data will be provided in fully anonymized form in accordance with UK NHS guidelines.
